# Assessment of coronary artery calcium by chest CT compared with EKG-gated cardiac CT in the multicenter AIDS cohort study

**DOI:** 10.1371/journal.pone.0176557

**Published:** 2017-04-28

**Authors:** Divay Chandra, Aman Gupta, Joseph K. Leader, Meghan Fitzpatrick, Lawrence A. Kingsley, Eric Kleerup, Sabina A. Haberlen, Matthew J. Budoff, Mallory Witt, Wendy S. Post, Frank C. Sciurba, Alison Morris

**Affiliations:** 1 Department of Medicine, University of Pittsburgh, Pittsburgh, Pennsylvania, United States of America; 2 Department of Radiology, University of Pittsburgh, Pittsburgh, Pennsylvania, United States of America; 3 Department of Epidemiology, University of Pittsburgh, Pittsburgh, Pennsylvania, United States of America; 4 Department of Medicine, University of California at Los Angeles, Los Angeles, California, United States of America; 5 Department of Epidemiology, Johns Hopkins University, Baltimore, Maryland, United States of America; 6 Department of Medicine, Johns Hopkins University, Baltimore, Maryland, United States of America; Fundacao Oswaldo Cruz, BRAZIL

## Abstract

**Rationale:**

Individuals with HIV are at increased risk for coronary artery disease (CAD). Early detection of subclinical CAD by assessment of coronary artery calcium (CAC) may help risk stratify and prevent CAD events in these individuals. However, the current standard to quantify CAC i.e. Agatston scoring requires EKG-gated cardiac CT imaging.

**Objective:**

To determine if the assessment of CAC using non-EKG-gated chest CT and the Weston scoring system is a useful surrogate for Agatston scores in HIV-infected and HIV-uninfected individuals.

**Methods and measurements:**

CAC was assessed by both the Weston and Agatston score in 108 men enrolled in the Multicenter AIDS Cohort Study.

**Results:**

Participants were 55.2 (IQR 50.4; 59.9) years old and 62 (57.4%) were seropositive for HIV. Inter-observer agreement (*r*_*s*_ = 0.94, κ = 90.0%, *p*<0.001, *n* = 21) and intra-observer agreement (*r*_*s*_ = 0.95, κ = 95.2%, *p*<0.001, *n* = 97) for category of Weston score were excellent. Weston scores were associated with similar CAD risk factors as Agatston scores (age, race, HDL cholesterol level, all *p*<0.05) in our cohort. There was excellent correlation (*r*_*s*_ = 0.92, *p*<0.001) and agreement (κ_w_ = 0.77, *p*<0.001) between Weston and Agatston scores.

**Conclusions:**

This study is the first to examine calcium scoring using chest CT in HIV-infected individuals and to independently validate the Weston score as a surrogate for the Agatston score. In clinical or research settings where EKG-gated cardiac CT is not feasible for the assessment of coronary calcium, Weston scoring by using chest CT should be considered.

## Introduction

Coronary artery disease (CAD) is among the top five causes of death in HIV-infected individuals and is becoming increasingly prevalent as these individuals live longer with modern anti-retroviral therapy [1–3]. Early detection of subclinical CAD may help risk stratify individuals with HIV and allow initiation of aggressive risk factor modification in high-risk individuals [4].

Subclinical CAD is commonly assessed by measurement of coronary artery calcium. Coronary artery calcium scores correlate with the extent of CAD measured by histology, intra-vascular ultrasound, and angiography [5–8] and add incremental prognostic information to clinical risk prediction models such as the Framingham score [9, 10]. However, assessment of coronary artery calcium traditionally requires EKG-gated cardiac CT and image analysis using proprietary cardiac imaging software (Agatston scoring) [11]. The limited availability and cost are prohibitive in many clinical and research settings. In contrast, routine chest CT imaging has proliferated in recent years and may provide a more feasible alternative to cardiac CT for assessment of coronary calcium.

Recently, a technique to quantify coronary artery calcium using routine chest CT has been described i.e. the Weston score [4]. Good agreement between Weston and Agatston scores has been reported in one study in an HIV-uninfected cohort [4]. However, these findings have not been independently replicated and the Weston score has not been examined in those with HIV. In the current study, we assessed the agreement between Weston vs. Agatston scores in HIV-infected and HIV-uninfected men enrolled in the Multicenter AIDS Cohort Study (MACS).

## Methods

### Study cohort

We included 108 participants enrolled in the Multicenter AIDS Cohort Study (MACS). This cohort is a prospective study of the natural and treated histories of HIV-1 infection in homosexual and bisexual men conducted in Baltimore, Chicago, Pittsburgh and Los Angeles. Men were ≥18 years in age and did not have clinical AIDS at study enrollment (according to the Centers for Disease Control and Prevention's 1987 definition). Other details regarding the MACS cohort have been reported elsewhere [12]. Study participants were selected for inclusion in the present analysis if they underwent both EKG-gated cardiac CT and non-EKG-gated chest CT within two years of each other as part of unrelated sub-studies in Pittsburgh and Los Angeles [13, 14]. The mean gap in time between the CT scan for the Weston vs. Agatston score was 21.8 ± 400.3 days, and for 50.9% of participants the two CT scans were performed within a year of each other.

The study was approved by the University of Pittsburgh Institutional Review Board and the University of Los Angeles Institutional Review Board. All participants provided written informed consent.

### Coronary artery calcium

#### Weston score

The chest CT examination was performed without cardiac gating, radiopaque contrast, tube current modulation, or iterative reconstruction algorithms at the Universities of Pittsburgh and University of California Los Angeles. Participants were scanned in the supine position at end-inspiration. A single GE Lightspeed VCT 64-detector scanner was used at the University of Pittsburgh and three Siemens 64-detector scanners were used at the University of California Los Angeles (see S1 Table: Image acquisition parameters for chest CT).

Weston scoring was performed by an internist without formal training in cardiac imaging after a teaching session using 20 sample cases. The four coronary arteries (left main, left anterior descending, right coronary, and left circumflex) were scored for the extent of calcification using a 4-point scale: 0 = no calcium, 1 = one pixel of calcium, 2 = more than one pixel but not enough to classify as 3, and 3 = “hard” calcium as evidenced by blooming artifact as described previously [4]. The branches of the major epicardial coronary arteries such as the obtuse marginal and diagonal arteries were not scored. Summed for the 4 arteries, the total Weston score ranged from 0 to 12 with increasing amounts of coronary calcium. The reader was blinded to other data on the subject, including their Agatston scores, and scored each CT scan in approximately one minute.

Inter-observer agreement was assessed by comparing Weston scores performed by the internist and by a radiologist specializing in cardiac imaging for 21 scans from an independent dataset with wide variability in the extent of coronary calcification. Intra-observer variability was assessed by blinded rescoring of 97 CT scans from an independent dataset by the internist at a minimum 3-month interval.

#### Agatston score

EKG-gated cardiac CT was performed as described previously [15]. In brief, CT scanning was performed using a 64‐slice multidetector CT with prospective ECG triggering protocols, which minimized radiation exposure (except when the heart rate was too fast or irregular). CT images were transferred to the core CT reading center (Biomedical Research Institute at Harbor‐UCLA, Los Angeles, CA) and were analyzed and interpreted by experienced readers who were blinded to participant characteristics and HIV serological status. Calcified atherosclerotic plaque was defined as any structure with attenuation >130 HU visualized separately from the intravascular lumen, identified visually in at least 2 independent planes. Agatston scores were calculated using the using the area density equation as described previously [11]. Coronary artery calcium scores were divided into four categories due to their highly skewed distribution per convention (none = 0, mild = 1–100, moderate = 101–400, and severe>400) [11].

### Cardiovascular risk factors

Hypertension was defined as a measured systolic blood pressure ≥140 mm Hg or diastolic blood pressure ≥90 mmHg, or use of anti-hypertensive medication with a report of a prior diagnosis of hypertension. Hyperlipidemia was defined by the lipid profile (fasting total cholesterol ≥ 200 mg/dl or LDL cholesterol ≥130 mg/dl or HDL < 40 mg/dl or triglycerides ≥150 mg/dl), or use of medication for hyperlipidemia with a self report of a prior diagnosis of hyperlipidemia. Similarly, diabetes was defined based on hemoglobin A1C ≥6.5 or fasting blood glucose ≥126 mg/dL, or reported use of medication for previously diagnosed diabetes. Lipid and glucose measurements were performed by the Heinz Nutrition Laboratory at the University of Pittsburgh Graduate School of Public Health as described previously [16, 17]. Measurements performed on blood samples collected closest to the date of the EKG gated CT scan were used; in all instances this was within the year preceding the CT scan or within the two weeks following the CT scan. Levels of C-reactive protein (CRP) were measured by means of a highly sensitive nephelometric assay using a monoclonal antibody to CRP coated on polystyrene beads with a lower limit of detection of 0.2 mg/L (Dade Behring, Marburg, Germany) as described previously [18].

Smoking history and race/ethnicity were self-reported, while BMI was measured by study personnel. Framingham 10-year risk was calculated based on cardiovascular risk profile as described previously [19]. Individuals with <10% risk were considered low risk, 10–20% as intermediate risk, and >20% as high risk.

### HIV status

HIV serostatus was defined on the date of the visit for the cardiac CT scan. CD4 cell count, HIV viral level, and use of combination antiretroviral therapy (ART) were assessed in HIV-infected individuals as described previously [12].

### Statistical analyses

Receiver operating characteristic (ROC) curves were used to identify the optimal cutoff for the Weston score that agreed with the established cutoff of 100 and 400 for defining categories of Agatston scores in the entire cohort. Inter- and intra-observer agreement for Weston scores was then tested using the weighted Kappa statistic (κ_w_). Participant characteristics were compared between those with or without any CAC per the Weston or Agatston score. Correlation between Weston and Agatston scores was tested using Spearman correlation coefficients (*r*_*s*_), while agreement between category of Weston vs. Agatston score was testing using the weighted Kappa statistic. Analyses were repeated by HIV status.

Chi-square test was used to compare categorical variables, while the Wilcoxon rank sum test was used for continuous variables. Analyses were performed using Stata MP Version 13.1 (StataCorp, College Station, TX). A two-tailed *p*<0.05 was defined as statistically significant.

## Results

### Participant characteristics

The median age of participants was 55.2 (IQR 50.4; 59.9) years, and most were non-Hispanic white (Table 1). 62 participants (57.3%) were HIV-infected, almost all of whom were on ART therapy with relatively preserved CD4 cell counts and low HIV viral levels. Hyperlipidemia was quite common in the overall cohort and most individuals were at low to intermediate Framingham 10-year risk.

**Table 1:** Characteristics of study participants stratified by presence/absence of coronary artery calcium defined by Weston or Agatston scores (median (IQR) for continuous variables and proportion for categorical variables).

Variable	All	Weston score		*p*	Agatston score		p*
		No CAC	Yes CAC		No CAC	Yes CAC	
*n*	108	47	61		44	64	
Age, years	55.2 (50.4; 59.9)	53.4 (48.3; 58.7)	57.6 (52.2; 61.3)	**0.02**	52.1 (47.8; 56.5)	57.7 (53.4; 62.1)	**<0.001**
Race (%)				0.08			**0.01**
Non-Hispanic White	86 (79.6)	33 (75.0)	53 (88.3)		29 (70.7)	57 (90.5)	
Other	18 (16.7)	11 (25.0)	7 (11.7)		12 (29.3)	6 (9.5)	
**HIV status**							
Seropositive (%)	62 (57.4)	28 (59.6)	34 (55.7)	0.69	26 (59.1)	36 (56.2)	0.77
CD4 count (*n* = 62, cells/mL)	711.5 (560.0; 955.0)	721.0 (587.0; 892.0)	693.0 (558.0; 980.0)	0.68	726.0 (587.0; 892.0)	693.0 (556.0; 984.0)	0.80
Viral load (*n* = 62, copies/mL)	49.0 (19.0; 49.0)	44.0 (19.0; 49.0)	49.0 (19.0; 49.0)	0.75	41.5 (19.0; 49.0)	49.0 (19.0; 49.0)	0.63
ART (*n* = 62, %)†	58 (53.7)	25 (53.2)	33 (54.1)	0.92	23 (52.3)	35 (54.7)	0.80
**CVD risk factors**							
Current smoking (%)	29 (26.9)	11 (23.4)	18 (29.5)	0.44	11 (25.0)	18 (28.1)	0.76
Pack years of smoking	2.8 (0.0; 22.0)	0.8 (0.0; 17.3)	5.3 (0.0; 29.0)	0.14	0.7 (0.0; 17.2)	4.5 (0.0; 29.5)	0.17
BMI (kg/m2)	26.5 (23.8; 30.5)	25.8 (23.4; 29.4)	26.8 (24.2; 32.1)	0.06	25.8 (23.5; 28.0)	26.8 (24.2; 32.1)	0.05
Hypertension (%)‡	54 (50.0)	22 (48.9)	32 (58.2)	0.35	19 (45.2)	35 (60.3)	0.14
Diabetes mellitus (%)§	10 (9.2)	5 (11.1)	5 (9.3)	0.76	5 (11.6)	5 (8.9)	0.65
Hyperlipidemia (%)**	87 (80.5)	37 (78.7)	50 (84.7)	0.45	34 (77.3)	53 (85.5)	0.26
Total cholesterol (mg/dL)	184.0 (159.0; 204.0)	186.0 (159.0; 204.0)	182.0 (159.0; 207.0)	0.53	185.5 (158.0; 205.0)	182.5 (160.0; 203.0)	0.70
HDL cholesterol (mg/dL)	46.0 (37.5; 54.5)	50.3 (38.0; 65.9)	42.9 (37.0; 50.2)	**0.02**	51.5 (40.4; 65.9)	42.7 (36.9; 50.4)	**0.003**
LDL cholesterol (mg/dL)	107.0 (86.0; 124.5)	112.0 (86.0; 130.0)	105.0 (84.0; 122.0)	0.49	109.5 (84.5; 130.5)	106.5 (86.0; 122.0)	0.90
Triglycerides (mg/dL)	124.0 (82.0; 194.0)	109.5 (74.0; 162.0)	130.0 (87.0; 216.0)	0.07	105.0 (74.0; 152.5)	134.0 (88.0; 217.0)	**0.02**
Hemoglobin A1c (%)	5.5 (5.3; 5.8)	5.5 (5.3; 5.8)	5.6 (5.3; 5.8)	0.60	5.5 (5.3; 5.8)	5.6 (5.3; 5.8)	0.51
Fasting glucose (mg/dL)	97.0 (90.0; 104.0)	96.0 (90.0; 101.0)	98.0 (91.0; 109.0)	0.22	95.0 (89.0; 100.0)	98.0 (92.0; 108.0)	0.05
C-reactive protein (mg/dL)	1.0 (0.6; 2.5)	0.9 (0.5; 2.2)	1.2 (0.6; 2.7)	0.44	1.1 (0.6; 2.3)	1.0 (0.5; 2.6)	0.78
Framingham 10-yr risk				0.16			0.10
Low (<10%)	69 (65.1)	33 (70.2)	36 (61.0)		33 (75.0)	36 (58.1)	
Intermediate (10–20%)	29 (27.4)	13 (27.7)	16 (27.1)		10 (22.7)	19 (30.6)	
High (>20%)	8 (7.5)	1 (2.1)	7 (11.9)		1 (2.3)	7 (11.3)	

* *p* values are from Wilcoxon rank-sum test for continuous variables, and from chi-square test for categorical variables

^†^ ART = combination antiretroviral therapy

^‡^ Measured blood pressure ≥140 systolic or ≥90 diastolic, or use of antihypertensive medication with prior diagnosis of hypertension

^§^ Hemoglobin A1C≥6.5 or fasting blood glucose ≥126 mg/dL or participant report of use of medication for diabetes with prior diagnosis of diabetes

** Fasting total cholesterol ≥ 200 mg/dl or LDL cholesterol ≥130 mg/dl or HDL < 40 mg/dl or triglycerides > = 150 mg/dl, or participant report of the use of medication to treat dyslipidemia with prior diagnosis of hyperlipidemia

### Optimal cut-points for Weston score

**Fig 1:**
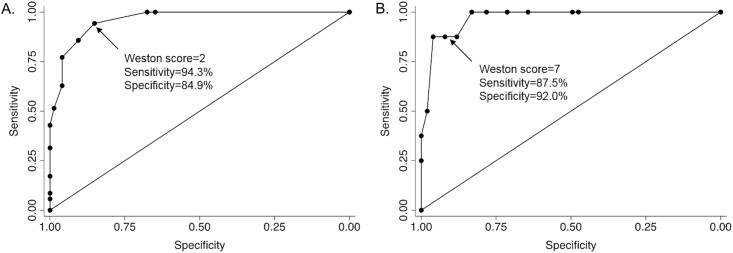
ROC curves for the selection of the optimal cut point for the Weston score that corresponds to the established cut point of 100 (panel A) and 400 (panel B) for the Agatston score.

Receiver operating curves suggested that a cut-point of 2 for the Weston score corresponded optimally to the established cut point of 100 for the Agatston score (sensitivity 94.3% and specificity 84.9%, Fig 1A). Similarly, a cut point of 7 for the Weston score corresponded optimally to the established cut point of 400 for the Agatston score (sensitivity 87.5% and specificity 92.0%, Fig 1B). Therefore, the Weston score was categorized as follows: 0, 1–2, 3–7, and >8.

### Inter- and intra-observer agreement for Weston score

Inter-observer agreement for category of total Weston score was excellent (κ_w_ = 90.0%, *r*_*s*_ = 0.94, both *p* <0.001, *n* = 21, Table 2). Inter-observer agreement was highest for calcium scores for the left anterior descending artery and lowest for those for the left main coronary artery (Table 2). Intra-observer agreement (κ_w_ = 95.2%, *r*_*s*_ = 0.95, both *p* <0.001, *n* = 97) was also excellent.

**Table 2:** Inter observer agreement for Weston coronary artery calcium score, n = 21 scans.

	κ_w_
**Total Weston Score**	0.90
**Score for each coronary artery**	
Left main	0.79
Left anterior descending	0.92
Right coronary	0.86
Left circumflex	0.82

### Correlates of Weston vs. Agatston scores

The majority of participants had detectable coronary artery calcium by either the Weston or Agatston score (Table 1). Both coronary artery calcium scores were associated with similar participant characteristics and risk factors including age and race. HIV status was not associated with coronary calcium assessed by either method in unadjusted analysis. Similarly, among HIV-infected individuals, CD4 cell counts, HIV viral levels, or use of ART were not associated with presence/absence of coronary artery calcium assessed by either method. The presence of coronary calcium by both scoring systems was associated with similar CAD risk factors (Table 1). Specifically, both scores had statistically significant association with low HDL cholesterol level and marginally significant associations with high BMI and high triglyceride level.

### Correlation between Weston and Agatston scores

**Fig 2:**
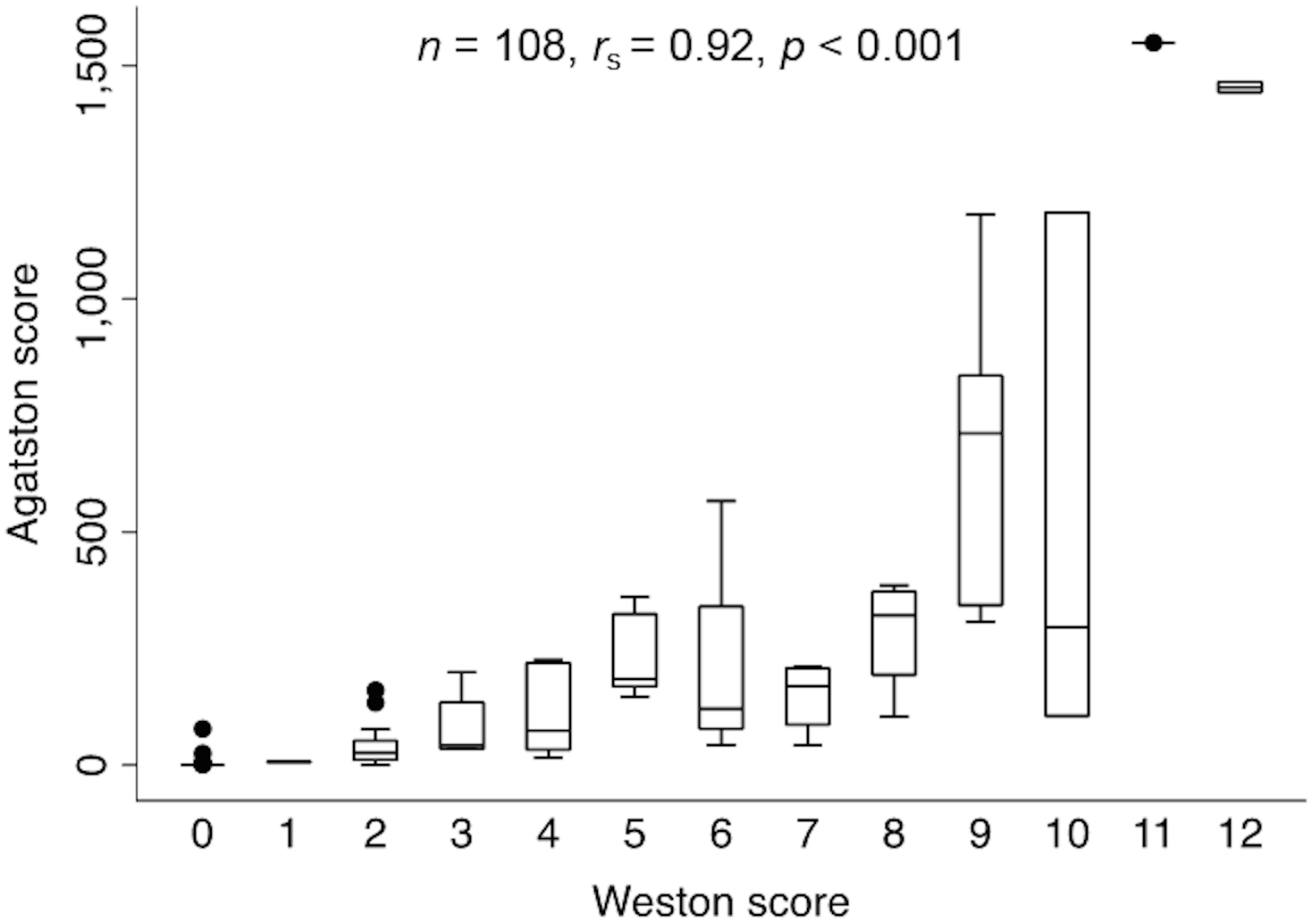
Correlation between Weston and Agatston scores performed on 108 individuals.

There was excellent correlation between Weston and Agatston scores (*r*_*s*_ = 0.92, *p*<0.001, Fig 2). The correlation was also excellent in the HIV-infected (*r*_*s*_ = 0.94, *p*<0.001) and HIV-uninfected individuals (*r*_*s*_ = 0.88, *p*<0.001).

### Agreement between categories of Weston and Agatston scores

For the presence/absence of coronary calcium, there was excellent agreement between Weston and Agatston score in all participants (κ_w_ = 0.87, *p*<0.001). In 101 participants (93.2%), both scores agreed about the presence/absence of CAC. Among the remaining 7, the Weston score was 0 while the Agatston score was slightly greater than 0 in 6 individuals (median 4.5, IQR 1.6–24.6), and the Agatston score was 0 while the Weston score was 2 i.e. in the mild category in 1 individual. Results were similar in HIV-infected (κ_w_ = 0.87, *p*<0.001) and HIV-uninfected individuals (κ_w_ = 0.86, *p*<0.001).

There was excellent agreement between category of Weston and Agatston score in all participants (κ_w_ = 0.77, *p*<0.001, Table 3). In 73.1% of cases, participants were classified in the same category of coronary artery calcium. In all cases of disagreement, participants were either in one higher or lower category of calcium score. The agreement appeared to be stronger in HIV-infected (κ_w_ = 0.82, *p*<0.001, Table 3) individuals where 74.2% were classified into the same category of calcium score compared with HIV-uninfected individuals (κ_w_ = 0.68, *p*<0.001, Table 3) where 65.2% of participants were in the same category for both scores.

**Table 3:** Comparison of category of coronary artery calcium defined by Weston compared with Agatston scores. The categories for Agatston score are: none = 0, mild = 1–100, moderate = 101–400, and severe>400. Categories for Weston score are: none = 0, mild = 1–2, moderate = 3–7, and severe = 8–12.

Weston score	Agatston score in all comers κ_w_ = 0.77, *p* < 0.001, *n* = 108				Total	Agatston score among HIV seropositive men κ_w_ = 0.82, *p* < 0.001, *n* = 62				Total	Agatston score among HIV seronegative men κ_w_ = 0.68, *p* < 0.001, *n* = 46				Total
	None	Mild	Moderate	Severe		None	Mild	Moderate	Severe		None	Mild	Moderate	Severe	
None	**42**	5	0	0	47	**25**	3	0	0	28	**17**	2	0	0	19
Mild	2	**13**	2	0	17	1	**8**	1	0	10	1	**5**	1	0	7
Moderate	0	11	**17**	1	29	0	5	**9**	0	14	0	6	**8**	1	15
Severe	0	0	8	**7**	15	0	0	3	**4**	10	0	0	5	**0**	5
**Total**	44	29	27	8	**108**	26	16	13	7	**62**	18	13	14	1	**46**

## Discussion

We compared coronary artery calcium assessment by non-EKG gated chest CT (Weston score) vs. EKG-gated cardiac CT (Agatston score). We identified 2 and 7 as optimal cut points for the Weston score that agreed with established cut points for 100 and 400 for the Agatston score. Also, we found excellent intra- and inter-observer agreement for Weston scores. Further, Weston scores were associated with similar cardiovascular risk factors as Agatston scores. Finally, there was excellent correlation between Weston and Agatston score, as well as excellent agreement for presence/absence of coronary calcium and for categories of coronary calcium between the Weston and Agatston score. This study is the first to provide independent validation of the Weston score as a useful surrogate for Agatston scores, and also the first to examine coronary artery calcium scoring using non-EKG-gated CT in HIV-infected individuals.

Detection of CAD at the subclinical stage may open a valuable window of opportunity to institute aggressive risk factor modification e.g. lipid lowering to mitigate the high morbidity and mortality from CAD in individuals with HIV. There are no blood tests that can reliably diagnose subclinical CAD. Resting and ambulatory EKGs suffer from a high prevalence of non-specific abnormalities limiting their utility in screening for CAD [20, 21]. Cardiac stress testing can provide indirect evidence for CAD by assessing for ischemia due to flow limiting stenosis; however, stress tests perform better in those with suspected CAD rather than in asymptomatic individuals in whom their sensitivity and specificity is suboptimal [22, 23]. Further, there is variability in the modality and interpretation of test results and the cost is considerable.

Coronary artery calcium scoring can directly identify the presence of CAD [5–8]. Calcium scores add prognostic information to the Framingham risk score and other risk prediction equations that are the foundation of preventive cardiology [19]. The 2010 ACCF/AHA guidelines gave the recommendations that “Measurement of CAC is reasonable for cardiovascular risk assessment in asymptomatic adults at intermediate risk (10% to 20%10-year risk, level of evidence: B, Class IIa) and “Measurement of CAC may be reasonable for cardiovascular risk assessment in persons at low to intermediate risk (6% to 10% 10-year risk, level of evidence B, Class IIb)”[24]. Notably, the individuals with and without HIV included in the current report were mostly at low and intermediate risk (Table 1).

The most common modalities used to assess coronary calcium include Agatston scoring and CT coronary angiography. These tests require specialized equipment (either an EBCT or a modern MDCT) that may not be readily available, especially in low-income countries. In contrast, non-EKG-gated chest CT scans are ubiquitous and commonly performed in clinical settings in developed countries, and may be the only modality available in low-income countries with high prevalence of HIV. Radiology reports for routine chest CT performed for other purposes increasingly make note of the presence and extent of coronary artery calcium [4]. Use in research cohorts has also increased greatly due to easy availability, low cost, and the ability to quantify pathology in diverse organ systems (lung, bone, muscle, fat, and liver) with one study procedure.

In HIV-uninfected individuals, prior studies have assessed coronary artery calcium from non-EKG gated CT either by visual methods or by Agatston score. Studies that have performed Agatston scoring have reported excellent correlation with Agatston scores from EKG gated scans obtained on the same individuals. For example, Hughes-Austin et al reported excellent correlation between Agatston scores from non gated vs. EKG-gated CT scans (*r* = 0.93, *n* = 4544). Similarly, Arcadi et al reported excellent correlation between Agatston from non gated vs. gated scans (*r* = 0.98, *n* = 66). For visual score, Azour et al. reported *r*_*s*_ = 0.81 between ordinal visual scores from non EKG gated CT and Agatston scores from EKG-gated CT (*n* = 222). Similary, Krish et al reported excellent correlation between Weston scores from non EKG gated CT compared with Agatson scores from EKG-gated CT (*r* = 0.83, *p*<0.001, *n* = 163). In a 2013 meta-analysis that included 5 studies comparing coronary artery calcium measurement from non gated (either visual score or Agatston score) vs. EKG-gated CT, the pooled agreement for categories of calcium score was high (κ = 0.89, 95% CI 0.83–0.95). In addition, independent studies have found that calcium scores from non-EKG CT predict survival and long-term outcomes irrespective of the method used for quantification [25–27]. Therefore, this is significant evidence that non-EKG gated CT can provide useful information about the extent and prognosis from coronary artery calcification in those without HIV.

Our study was the first to examine the utility of calcium scoring of non EKG-gated scans in individuals with HIV. We decided to implement the Weston score because it does not require the software needed for measuring Agatson scores. The prior report comparing Weston to Agatston scores in individuals without HIV also found excellent inter- and intra-observer agreement for Weston scores, and good agreement between Weston and Agatston scores [4]. The optimal cut points identified for categorizing the Weston score in the prior report were the same as in our investigation (2 and 7) and are therefore independently validated. 44 of 108 individuals in our cohort had an Agaston score of 0 indicating an excellent ten-year prognosis. 42 of these 44 individuals also had a Weston score of 0, suggesting that the Weston score can provide important prognostic information.

Our study has a number of limitations. First, Weston scoring is performed visually, therefore, results may be more subjective than Agatston scores where visual interpretation has a minimal role [11]. Second, our EKG-gated and non-EKG-gated CT scans were not performed on the same day, but were done within two years of each other which may have introduced error. However, this difference in timing would lead to more conservative estimates of agreement compared to scans performed closer to each other. Third, our study was limited to men and may not be generalizable to CT scans in women. Also, we did not find an association between coronary calcium and HIV serostatus. However, we previously demonstrated associations between HIV serostatus and non-calcified plaque detected by coronary CT angiography and a higher incidence of coronary calcium in HIV- infected men compared to uninfected men in the larger MACS cohort [15, 28]. Further, the CT scans used for Weston scoring were acquired at modest radiation dose (100 mAs) and results may not be generalizable low dose CT scans obtained for lung cancer screening or to higher dose high resolution CT scans obtained for clinical indications. Also, our study did not have data to demonstrate that Weston scores predict mortality or cardiac events in those with HIV. Finally, it is unclear if Weston scores will be more or less sensitive to longitudinal change compared with Agatston scores, although serial calcium assessments are not recommended by the 2010 ACCF/AHA guidelines due to lack of data that they lead to improved outcomes or changes in therapeutic decision making [24].

Our results suggest that Weston may be a good surrogate for Agatston scores and should be considered in clinical or research settings where Agatston scoring is not feasible. The agreement between Weston and Agatston scores should be further examined in cohorts of individuals with HIV that include women.

## Supporting information

S1 Table Image acquisition parameters for chest CT. (DOCX)
