# Supplementary material for: Assessment of coronary artery calcium by chest CT compared with EKG-gated cardiac CT in the multicenter AIDS cohort study
Source: PLoS One. 2017 Apr 28;12(4):e0176557. doi: 10.1371/journal.pone.0176557 (PMC5409142; doi:10.1371/journal.pone.0176557)
Supplement: S1 Table — (DOCX) [file pone.0176557.s001.docx]

**S1 Table:** Image acquisition parameters for chest CT.

|  | **Chest CT** | |
| --- | --- | --- |
|  | Pittsburgh | Los Angeles |
| **Model** | GE LightSpeed VCT | Siemens Sensation 64  Definition  Definition Flash |
|  |  |  |
|  |  |  |
| **Acquisition** |  |  |
| Acquisition mode | Helical | Spiral |
| Detector configuration | 64 x 0.625 | 64 x 0.6 |
| Energy (kVp) | 120 | 120 |
| Effective exposure, max, (mAs) | n/a | 100 (125) |
| Tube current, max, (mA) | 200 (250) | n/a |
| Gantry rotation time (sec) | 0.5 | 0.5 |
| Pitch | 1.375 | 1.100 |
| Tube current modulation | Auto & Smart mA - off | Care Dose - off |
| **Image Reconstruction** |  |  |
| Iterative reconstruction | ASIR - off | IRIS – off |
| Kernel | Standard | B31f |
| Thickness (mm) | 2.5 | 3.0 |
| Interval (mm) | 2.5 | 3.0 |
| Image matrix | 512 x 512 | 512 x 512 |
